# Improvement in Thermal Conductivity in UV-Curable Polymer Composites via h-BN and Graphite Hybrid Fillers for DLP 3D Printing

**DOI:** 10.3390/ma19112304

**Published:** 2026-05-29

**Authors:** Marco Fortunato, Cristina Stifani, Alessandra Fava, Maria Rita Mancini, Ugo De Angelis, Giuseppe De Santis, Giuseppe Corallo, Daniele Mirabile Gattia

**Affiliations:** Department of Sustainability, Circularity, and Climate Change Adaptation of Production and Territorial Systems (SSPT), Research Centre of Casaccia, Italian National Agency for New Technologies, Energy and Sustainable Economic Development (ENEA), Via Anguillarese 301, 00123 Rome, Italy; cristina.stifani@enea.it (C.S.); alessandra.fava@enea.it (A.F.); ugo.deangelis@enea.it (U.D.A.); giuseppe.desantis@enea.it (G.D.S.);

**Keywords:** digital light processing (DLP), UV-curable polymer composites, hexagonal boron nitride (h-BN), graphite, thermal conductivity, percolation modeling

## Abstract

**Highlights:**

- DLP-printed h-BN/graphite composites with enhanced thermal conductivity.
- DLP printing for highly loaded thermally conductive composites.
- Percolation-based model predicts thermal conductivity vs. h-BN filler content.
- Interconnectivity index quantifies heat-transport pathway formation.
- Graphite enables tunable electrical and thermal conductivity in h-BN-based composites.

**Abstract:**

UV-curable polymer composites are attractive for fabricating complex components by digital light processing (DLP), but improving thermal transport while preserving printability remains challenging at high filler loadings. In this work, solvent-free UV-curable formulations filled with hexagonal boron nitride (h-BN) and h-BN/graphite hybrids were developed for DLP 3D printing using commercially available equipment. The effects of filler composition on viscosity, printability, microstructure, through-thickness thermal conductivity, electrical conductivity, and tensile behavior were investigated. Viscosity increased markedly with filler loading, yet reliable DLP printing was achieved up to 40 wt% h-BN through composition-dependent adjustment of build parameters. Thermal analysis supported negligible macroscopic sedimentation during printing, while optical and FE-SEM observations revealed generally uniform platelet dispersion, visible 50 μm layer stratification, and limited phase segregation in the hybrid systems. The through-thickness thermal conductivity increased from ~0.25 W/mK for the neat resin to ~1.95 W/mK at 40 wt% h-BN. At a fixed 20 wt% h-BN, graphite addition led to a smaller increase in thermal conductivity, up to ~1.16 W/mK, while increasing electrical conductivity and reducing mechanical performance. A phenomenological percolation-type model captured the thermal-conductivity trend of the h-BN series. Overall, h-BN-rich formulations provided the most effective route to enhance thermal conductivity while preserving electrical insulation.

## 1. Introduction

Polymer-based materials are widely used in electronics, sensors, photonics, and energy-related systems because of their low density, chemical stability, ease of processing, and broad tunability. However, their intrinsically low thermal conductivity, typically below 0.3 W/mK, remains a major limitation in applications requiring efficient heat dissipation, such as power electronics, wearable devices, and light-emitting systems, where heat accumulation can accelerate performance degradation and compromise reliability [1,2].

A common strategy to overcome this limitation is the incorporation of thermally conductive fillers into the polymer matrix, leading to thermally conductive polymer composites (TCPCs). Among the available fillers, hexagonal boron nitride (h-BN) is particularly attractive because it combines high intrinsic thermal conductivity, electrical insulation, chemical inertness, and good compatibility with polymer matrices [2,3,4]. Graphite is also of considerable interest owing to its high thermal conductivity, low cost, and wide commercial availability, making it a promising co-filler for multifunctional thermal-management systems [5,6,7]. More broadly, hybrid filler concepts based on ceramic and carbonaceous phases have been extensively explored to improve filler connectivity and facilitate the formation of more efficient heat-transfer pathways [8,9,10,11]. Despite these advantages, substantial improvements in thermal conductivity are often obtained only at high filler loadings, which can strongly increase viscosity, deteriorate mechanical performance, and narrow the processing window. This issue is particularly relevant for photopolymerization-based additive manufacturing, where the formulation must remain compatible not only with rheological constraints, but also with light penetration, curing depth, layer recoating, and interlayer adhesion. In digital light processing (DLP), these coupled effects become especially critical at increasing solids content because filler loading affects both resin mobility and optical response during layer-by-layer fabrication [1]. Recent studies have shown that highly efficient thermal transport can be achieved through engineered filler architectures, nano-interconnected networks, or hybrid conductive pathways that reduce interfacial thermal resistance and improve transport efficiency [9,12,13,14,15]. High through-plane thermal conductivity has also been reported in electrically insulating BN/polymer systems [9]. However, many of these high-performing strategies rely on tailored microstructures or processing routes that are not directly transferable to commercially relevant vat-photopolymerization workflows. At the same time, relatively few studies have examined, in an integrated manner, how filler composition affects viscosity, printability, microstructure, through-thickness thermal conductivity, electrical behaviour, and tensile response in solvent-free UV-curable formulations processable with standard DLP equipment. This gap is important because, for practical applications, the key issue is not only whether thermal conductivity can be increased, but under which conditions a useful balance can be maintained among thermal performance, electrical insulation, mechanical integrity, and processability. Within this framework, h-BN and graphite can play distinct and potentially complementary roles. h-BN is expected to enhance thermal transport while preserving electrical insulation, whereas graphite may introduce additional conductive pathways and modulate the electrical response, but also increase brittleness and reduce processing latitude. Therefore, the scientific interest of h-BN/graphite systems lies less in claiming a universal synergistic advantage and more in understanding the trade-offs that emerge when multifunctional transport behaviour is pursued in DLP-printable composites.

In this work, solvent-free UV-curable formulations filled with h-BN and h-BN/graphite hybrids were developed for DLP 3D printing using commercially available equipment. The aim of the study is fourfold: (i) to define the formulation and printability window as a function of filler loading; (ii) to correlate viscosity and microstructural features with the development of through-thickness heat-transfer pathways; (iii) to assess how graphite addition modifies the thermal, electrical, and mechanical response of h-BN-based composites; and (iv) to describe the composition-dependent thermal-conductivity trend of the h-BN series by means of a phenomenological percolation-type model. Rather than targeting record thermal conductivity through highly engineered architectures, this study focuses on a scalable DLP-compatible platform and on the process–structure–property relationships governing thermally enhanced UV-curable composites.

## 2. Materials and Methods

A commercial UV-curable acrylate-based photopolymer resin (High Clear Resin, Anycubic, Shenzhen, China) was used as the polymer matrix. Two thermally conductive fillers were selected: hexagonal boron nitride (h-BN, average particle size ~1 μm) and graphite flakes (Sigma-Aldrich, St. Louis, MO, USA, particle size < 20 μm). Prior to compounding, the powders were stored in sealed containers under ambient conditions. To reduce volatile adsorption and improve dispersion, the h-BN powder was thermally desorbed in a ventilated oven at 180 °C for 16 h and then cooled to room temperature in a desiccator. Graphite flakes were used as received, without further purification.

### 2.1. Samples Fabrication

**Figure 1:**
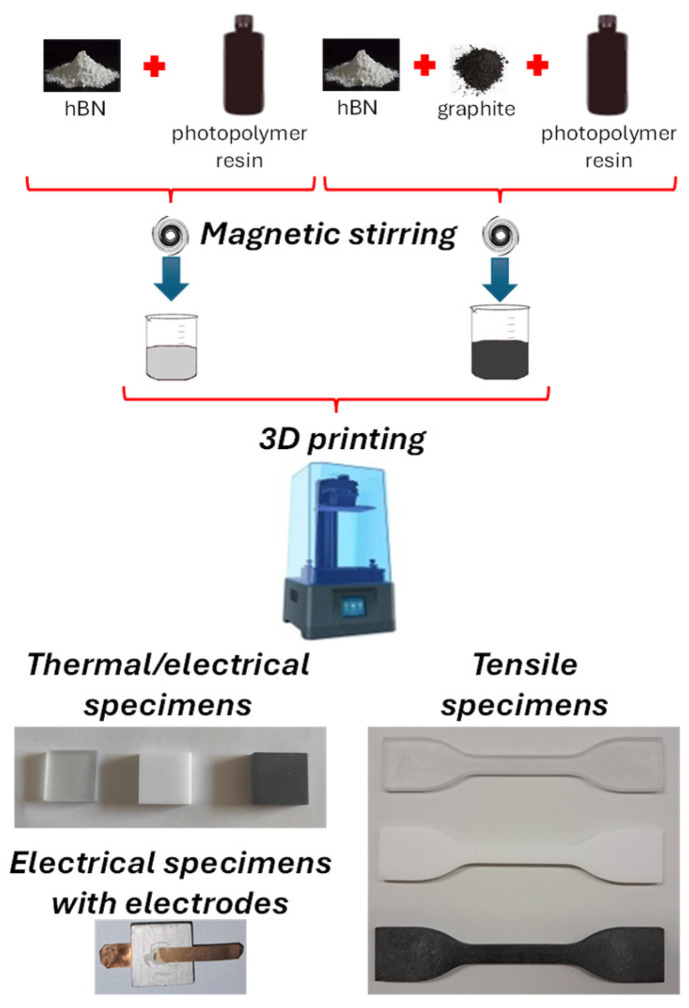
Schematic workflow for the preparation of h-BN and h-BN/graphite formulations and images of the printed specimens used for thermal/electrical and tensile testing.

Composite formulations were prepared by gradually adding the fillers to the UV-curable resin under reduced light exposure in order to avoid premature curing. Two composition series were investigated: (i) an h-BN series ranging from 5 to 40 wt% in 5 wt% increments; and (ii) a hybrid series based on 20 wt% h-BN with additional graphite contents of 0, 1, 2.5, 8, and 10 wt%. The fillers were introduced progressively into the resin during mixing to promote wetting and limit agglomeration. The suspensions were magnetically stirred for 1 h under ambient conditions (room temperature (RT) ≈ 23 °C; relative humidity (RH) ≈ 73%). A schematic overview of the preparation route and of the specimen geometries is shown in Figure 1.

For the subsequent analyses and modeling, the filler mass fractions (*w*) were converted into volume fractions (*φ*) using the density-based relation:

(1) $$\varphi =\frac{\sum_{i}\frac{w_{f,i}}{\rho_{f,i}}}{\frac{w_{m}}{\rho_{m}}+\sum_{i}\frac{w_{f,i}}{\rho_{f,i}}}\;$$

where *w_f_*_,*i*_ is the mass fraction of filler *i*, $w_{m}=1-\sum_{i}w_{f,i}$ is the matrix mass fraction, $\rho_{f,i}$ is the density of filler *i*, and *ρ*_*m*_ is the density of the matrix. For subsequent analyses and modeling, the following values were used: $\rho_{m}=1.15\;g/{cm}^{3}$, $\rho_{f\_h-BN}=2.10\;g/{cm}^{3}$, and $\rho_{f\_G}=2.25\;g/{cm}^{3}$. Here, $\rho_{f\_h-BN}$ denotes the density of the h-BN and $\rho_{f\_G}$ the density of the graphite.

### 2.2. DLP Printing and Post-Processing

**Figure 2:**
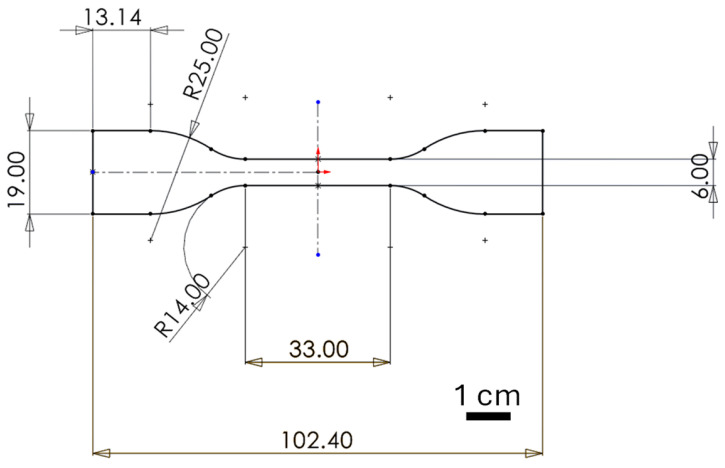
Geometry and dimensions of the dog-bone specimen used for tensile testing.

Composite specimens were fabricated using a commercial digital light processing (DLP) 3D printer (Anycubic Photon Ultra, Anycubic, Shenzhen, China) with a nominal layer thickness of 50 μm. Because both filler type and filler loading affect UV light penetration, curing behavior, and resin viscosity, the printing parameters were adjusted depending on composition. Starting from the neat resin and the manufacturer-recommended printing parameters, each formulation was tested sequentially with increasing filler loading. For each composition, the printing parameters were adjusted until defect-free samples could be obtained. Once a satisfactory parameter set was identified, printability was verified by producing three samples in three consecutive print runs. If printing failed, additional parameter sets were tested until successful fabrication was achieved or the formulation was considered non-printable under the investigated conditions. In particular, increasing filler loading required longer exposure of the initial layers, a higher number of bottom layers, and tuning of lift speed and settling delay in order to reduce the risk of resin starvation, under-curing, and layer delamination. Two specimen geometries were printed:

- Rectangular specimens with dimensions of (20 × 20 × 6) mm^3^ were used for thermal-conductivity and through-thickness electrical-conductivity measurements.
- Dog-bone specimens were used for tensile testing, according to the geometry shown in Figure 2: overall length 102.4 mm, grip width 19 mm, gauge width 6 mm, gauge length 33 mm, and shoulder radii of R25 and R14.

After printing, the parts were cleaned in isopropanol using an ultrasonic bath to remove uncured resin and were subsequently post-cured in a dedicated UV curing unit.

### 2.3. Characterization Methods

The viscosity of the uncured formulations was measured using a Fungilab Advance R rotational viscometer (Fungilab, Sant Feliu de Llobregat, Spain) equipped with a small-sample adapter (APM, test volume: 7.1 mL) and a TR8 spindle. Measurements were carried out at a constant temperature of 20 ± 0.5 °C. For each formulation, the spindle speed was adjusted to maintain the torque within the recommended range of 30–90%. Viscosity values were recorded after 15, 30, and 45 min, and only measurements falling within the acceptable torque interval were retained. For each formulation, the rotational speed was selected so as to keep the measured torque within the reliable operating range of the instrument. Therefore, the reported viscosity values were obtained under controlled and instrumentally reliable conditions, although no full rheological flow curve as a function of shear rate was determined in the present work.

Thermal analysis was performed by simultaneous thermogravimetric and differential thermal analysis (TG/DTA) using a Netzsch STA449C Jupiter instrument (NETZSCH-Gerätebau GmbH, Selb, Germany). Approximately 15 mg of material was placed in alumina pans and heated from 20 to 900 °C at a rate of 10 °C/min under an air flow of approximately 25 sccm. The residual mass at 900 °C was used to estimate the inorganic fraction remaining after matrix decomposition and to assess possible filler sedimentation during processing.

Morphological characterization was carried out by optical microscopy (Keyence VHX-X1, Keyence Corporation, Osaka, Japan) and field-emission scanning electron microscopy (FE-SEM, Zeiss Leo 1530, Carl Zeiss, Oberkochen, Germany). To minimize charging effects, cross-sections were sputter-coated with an approximately 5 nm thick Au layer prior to FE-SEM observation. SEM images were acquired at an accelerating voltage of 5 kV. The as-received fillers were also examined in order to verify their particle size and morphology.

Thermal conductivity was measured using a Hot Disk TPS 500s (Hot Disk AB, Gothenburg, Sweden) instrument in the transient plane source (TPS) configuration. A nickel double-spiral sensor (C5465, radius 3.2 mm) was sandwiched between two planar halves of each rectangular specimen. For each composition, 15 measurements were performed and averaged. The reported uncertainty corresponds to the standard deviation of the repeated measurements.

Through-thickness direct-current electrical conductivity was determined by depositing silver electrodes on the two opposite large faces of each rectangular sample and recording two-terminal current–voltage characteristics using a Keithley 6517B electrometer (Keithley Instruments Inc., Cleveland, OH, USA). The electrical conductivity was calculated from the measured resistance, sample thickness, and electrode area.

Mechanical properties were evaluated by tensile testing on DLP-printed dog-bone specimens. The tests were carried out on specimens with the geometry shown in Figure 2. The maximum tensile stress and elongation at break were used to assess the effect of filler loading and graphite addition on the mechanical response of the composites.

## 3. Results

The Results section is organized into four main parts: rheological and thermal analyses of the formulations, morphological characterization of fillers and printed composites, thermal and electrical transport properties, and the mechanical response of the printed materials.

### 3.1. Viscosity Measurements

**Figure 3:**
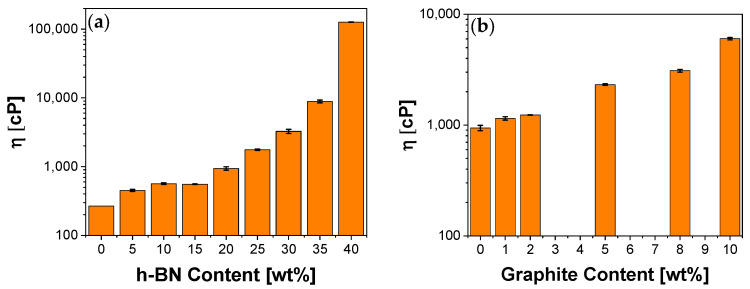
Viscosity (*η*) of uncured h-BN/resin formulations as a function of h-BN loading (**a**) and viscosity of resin containing 20 wt% h-BN as a function of graphite content (**b**).

The viscosity of the uncured formulations increased markedly with filler loading. As shown in Figure 3a, the neat resin exhibited a viscosity of 266 cP, which progressively rose to 126,185 cP at 40 wt% h-BN. This monotonic increase is consistent with the growing effective volume fraction of the dispersed platelets, enhanced filler–filler interactions at higher loading, and the resulting reduction in resin mobility. From a processing standpoint, these trends justified the use of composition-dependent DLP parameters, including longer exposure of the first layers and adjusted lift speed and settling delay at higher h-BN contents. The viscosity window up to 20 wt% h-BN remained compatible with standard vat photopolymerization, whereas higher filler contents required tighter control of the printing protocol to avoid resin starvation and incomplete curing. Hybrid h-BN/graphite formulations showed the same qualitative trend (Figure 3b). At a fixed h-BN content of 20 wt%, viscosity increased from 941 cP to 6022 cP with increasing graphite fraction. Low graphite additions (≤2 wt%) caused only modest thickening, whereas graphite contents above 5 wt% shifted the formulation into a markedly higher-viscosity regime. Although these mixtures remained printable, they required more careful control of exposure and settling conditions. In operational terms, printability was assessed on the basis of successful and reproducible fabrication of defect-free specimens after parameter optimization. The main observed failure modes at increasing filler loading were incomplete curing or under-cured layers, resin starvation due to insufficient resin refill, local delamination or poor interlayer bonding, and dimensional inaccuracies of the printed parts. Up to 40 wt% h-BN, these issues could be mitigated through composition-dependent adjustment of the build parameters. By contrast, for filler contents above 40 wt% h-BN, at least 10 different parameter sets were tested, but none enabled successful fabrication of acceptable samples. Therefore, 40 wt% h-BN was considered the upper practical printability limit within the investigated processing window. It should be noted that the viscosity measurements were performed by selecting the rotational speed so that the measured torque remained within the reliable operating range of the instrument. Since the DLP process investigated here involves slow resin reflow and relatively low relative motion during layer formation, the practical printability of the formulations is expected to depend mainly on their low-shear rheological response.

### 3.2. Thermal Analysis (TG/DTA)

**Figure 4:**
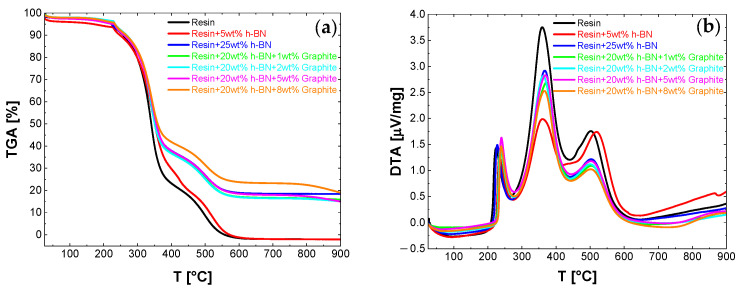
TGA curves (**a**) and DTA thermograms (**b**) for the neat resin, h-BN-filled composites (5–40 wt% h-BN), and hybrid h-BN/graphite systems (20 wt% h-BN with 0–10 wt% graphite).

Simultaneous TG/DTA analysis was used to verify whether the inorganic content of the cured printed parts was consistent with the nominal composition of the uncured blends and to assess the thermal events associated with matrix degradation. The TGA residual masses measured at 900 °C are summarized in Table 1. In all investigated formulations, the measured residual mass was in good agreement with the nominal filler content, with relative deviations below 5%. This result supports the absence of significant macroscopic sedimentation during formulation, printing, and post-curing. The visual appearance of the final residue was also consistent with the filler type, being white in h-BN-filled composites and grey in h-BN/graphite hybrids. Representative TGA curves and DTA thermograms are shown in Figure 4a and Figure 4b, respectively.

The DTA curves exhibited three main exothermic events, indicating staged oxidation/decomposition of the photopolymer matrix. Overall, the thermal analysis confirmed that the inorganic fraction remaining after degradation was consistent with the targeted filler loading and that no major filler segregation occurred during processing.

### 3.3. Morphological Characterization

**Figure 5:**
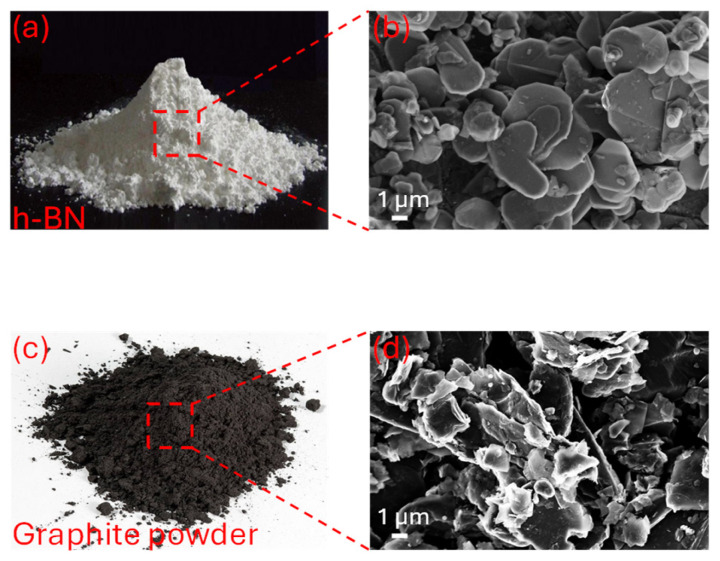
Image and FE-SEM micrographs of h-BN powder (**a**,**b**) and graphite powder (**c**,**d**).

The as-received fillers were first examined by optical microscopy and FE-SEM in order to verify their morphology and approximate particle size. As shown in Figure 5, the graphite flakes exhibited in-plane lateral dimensions below 20 μm, whereas the h-BN powder consisted of platelet-like particles with lateral dimensions of a few micrometers, in agreement with the supplier specifications.

**Figure 6:**
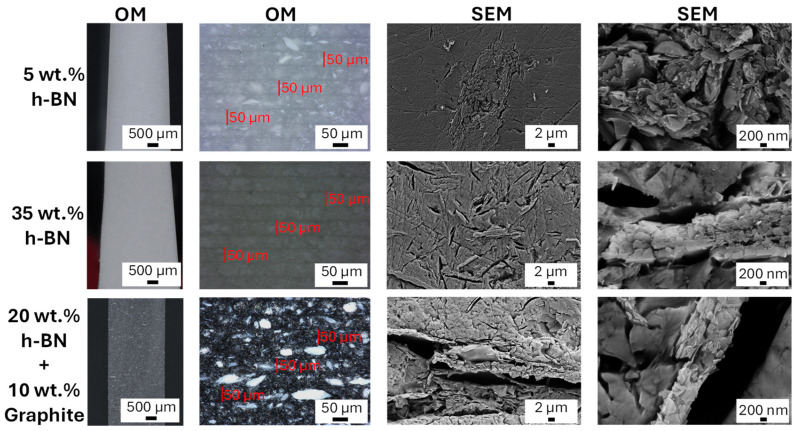
Optical microscopy and FE-SEM cross-sectional images, taken along the build direction (z direction), of DLP-printed composites containing 5 wt% h-BN, 35 wt% h-BN, and 20 wt% h-BN + 10 wt% graphite.

Representative OM and FE-SEM micrographs of selected printed composites are shown in Figure 6. The OM images clearly revealed the layered architecture produced by DLP printing, with a characteristic layer thickness of approximately 50 μm, consistent with the slicing parameter used during fabrication. In the h-BN-filled samples, the filler was generally well dispersed throughout the polymer matrix, although occasional micrometer-scale agglomerates were observed, especially at higher filler loading. In particular, agglomerates with typical dimensions of approximately 5–10 μm were observed in the h-BN-filled samples, whereas larger agglomerates, with dimensions of about 20–30 μm, were found in the h-BN/graphite formulations. Compared with the initial size of the h-BN particles, this result suggests that the presence of graphite may favor the formation of larger aggregates in the hybrid systems, possibly by acting as a local aggregation site for h-BN particles. In the hybrid formulations, graphite flakes appeared co-dispersed within the h-BN-containing matrix with limited phase segregation. The fracture-surface images also suggested good filler incorporation in the cured matrix, with local pull-out features observed in some regions. In addition, the cross-sectional optical and FE-SEM observations do not reveal obvious filler-rich/filler-poor gradients across the printed thickness, even in the higher-loading formulations, such as 35 wt% h-BN and the hybrid 20 wt% h-BN + 10 wt% graphite. This qualitative evidence is consistent with the TGA results and supports the absence of evident macroscopic sedimentation during printing.

### 3.4. Effect of Filler Concentration on the Thermal Conductivity

#### 3.4.1. Thermal Conductivity

**Figure 7:**
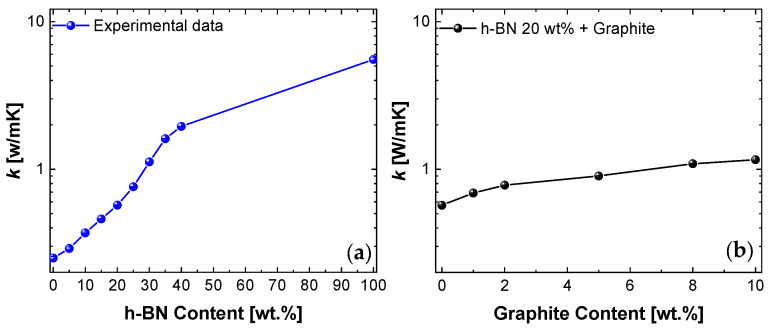
Through-thickness thermal conductivity of DLP-printed composites as a function of h-BN content (**a**) and as a function of graphite content at fixed h-BN loading (20 wt%) (**b**).

The through-thickness thermal conductivity of the printed composites increased with filler loading, as shown in Figure 7. For the h-BN-only series, the neat resin exhibited a thermal conductivity of approximately 0.25 W/mK. Upon increasing h-BN content, the thermal conductivity rose progressively, showing a mild increase at low filler content and a steeper rise above approximately 15 wt%. At 30 wt% h-BN, the thermal conductivity exceeded 1 W/mK, while at 40 wt% h-BN, it reached approximately 1.95 W/mK, corresponding to an almost eightfold enhancement over the neat resin (Figure 7a).

At a fixed h-BN loading of 20 wt%, graphite addition produced a further increase in thermal conductivity, from approximately 0.56 W/mK up to approximately 1.16 W/mK at 10 wt% graphite (Figure 7b). The largest incremental increase occurred at low graphite additions, whereas the improvement became more gradual at higher graphite fractions.

#### 3.4.2. Theoretical Model

To predict the thermal conductivity as a function of h-BN loading, we adopted a saturating percolation model, based on Chung’s model [16], which suitably reproduces the observed sigmoidal behaviour. Expressed in terms of the h-BN volume fraction *ϕ* (computed from mass fractions via Equation (1)), the effective thermal conductivity is modelled as:

(2) $$k\left(\Phi \right)=\;k_{m}+\left(k_{f}-k_{m}\right)\cdot \left(\frac{\Phi^{n}}{\Phi^{n}+\Phi_{c}^{n}}\right)$$

where *k*_*m*_ is the thermal conductivity of the neat resin; *k_f_* is the maximum conductivity achievable at high h-BN fractions; *ϕ* is the h-BN volume fraction; *ϕ*_*c*_ is a characteristic transition parameter (often referred to as a “percolation threshold” in sigmoidal formulations); and *n* is the parameter that controls the steepness of the sigmoidal transition. The model reproduces the experimental data under the following conditions:

- $\Phi_{c}=0.75$. This relatively high value should be interpreted as a phenomenological parameter of the sigmoidal fit rather than a strict geometrical percolation threshold. In fact, the experimentally observed change in slope occurs at h-BN loadings of a few tens of wt.%, where more effective through-thickness heat-transfer pathways start to develop. The need for relatively high filler loadings is plausibly associated with incomplete inter-particle contacts, limited real contact area between neighboring platelets, and agglomeration effects, together with interfacial thermal resistance and printing-induced microstructural discontinuities, which delay the formation of a well-connected conductive network across the sample thickness.
- n=2.29. The transition to the conductive regime is sharp; this value indicates that conductivity rises rapidly once the percolation threshold is exceeded.
- $k_{f}=8.45\;W/\mathrm{mK}$. This represents the upper limit of thermal conductivity theoretically attainable by the composite if a well-connected, continuous percolative network is established. This estimate is in good agreement with an independent measurement on a pellet of neat h-BN (~3 cm diameter, ~6 mm thickness) pressed in an autoclave, which exhibited a through-thickness thermal conductivity of ~6 W/mK.

**Figure 8:**
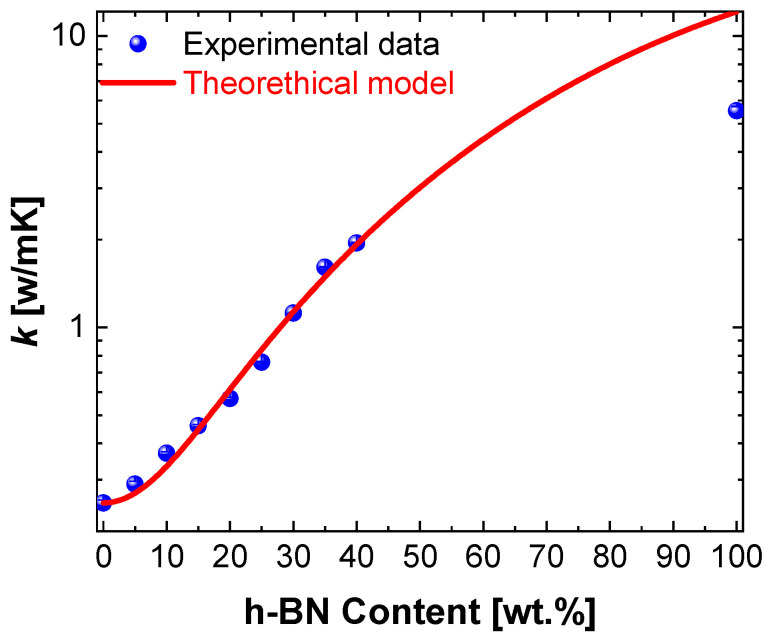
Through-thickness thermal conductivity of DLP-printed composites as a function of h-BN content: experimental data (symbols) and phenomenological model fit (solid line).

Figure 8 compares the experimental data (symbols) with the model predictions (solid line). The model captures well the experimental trend over the entire investigated range of h-BN contents.

#### 3.4.3. Interconnectivity Analysis

To estimate how effectively the filler phase forms continuous conductive pathways, we compare the measured thermal conductivity $k_{\mathrm{meas}}$ with the Hashin–Shtrikman (HS) bounds and defines a normalized interconnectivity index X∈[0,1] [17]:

(3) $$X=\frac{k_{meas-}k_{\mathrm{HS}}^{-}}{k_{\mathrm{HS}}^{+}-k_{\mathrm{HS}}^{-}}$$

where $k_{meas}$ is the measured thermal conductivity and $k_{HS}^{-}$ and $k_{HS}^{+}$ are the lower and upper Hashin–Shtrikman bounds, respectively. The corresponding expressions for the two-phase system are given in Equation (4).

(4) $$\left\{\begin{array}{c} k_{\mathrm{HS}}^{-}=k_{m}+\frac{\phi }{\frac{1}{\;k_{f}-k_{m}}+\frac{1-\phi }{3\;k_{m}}}\\ k_{\mathrm{HS}}^{+}=k_{f}+\frac{1-\phi }{\frac{1}{\;k_{m}-k_{f}\;}+\frac{\phi }{3\;k_{f}}} \end{array}\right.$$

where $k_{m}$ is the neat-resin thermal conductivity, $k_{f}$ is the effective thermal conductivity of the filler phase, and ϕ is the filler volume fraction (weight fractions converted using $\rho_{m}\approx 1.15\;{g/\mathrm{cm}}^{3}$, $\rho_{h-BN}\approx 2.10\;{g/\mathrm{cm}}^{3}$, $\rho_{G}\approx 2.25\;{g/\mathrm{cm}}^{3}$).

**Figure 9:**
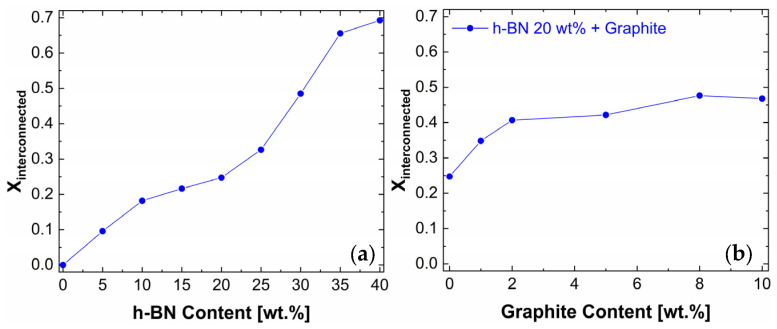
Interconnectivity index as a function of h-BN content (**a**) and as a function of graphite content at fixed h-BN loading (20 wt%) (**b**).

The interconnectivity index *X* increases with h-BN content: from ~0 at 0 wt% h-BN to ~0.7 at 40 wt% h-BN, indicating the progressive formation of continuous heat-conduction pathways (Figure 9a). At fixed h-BN: 20 wt%, adding graphite raises *X* from ~0.2 (0 wt% of graphite) to ~0.5 (10 wt% of graphite), suggesting that graphite flakes co-participate in the h-BN-driven network, with gains that begin to saturate beyond ~8 wt% (Figure 9b). Although the highest X value indicates a significantly developed network, it still remains below the upper Hashin–Shtrikman bound and is therefore not yet fully optimized. This behavior is consistent with incomplete inter-particle contact, limited real contact area between neighboring platelets, interfacial thermal resistance, local agglomeration, random platelet distribution, and possible microstructural discontinuities associated with the layer-by-layer DLP process. It should therefore be interpreted as a phenomenological comparative indicator rather than as a direct quantitative measure of the actual geometric connectivity of the filler phase.

### 3.5. Electrical Conductivity

**Figure 10:**
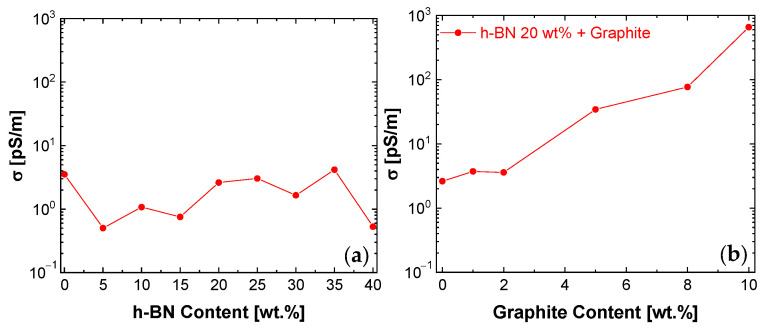
Through-thickness electrical conductivity as a function of h-BN content (**a**) and graphite content at fixed h-BN loading (20 wt%) (**b**).

The through-thickness DC electrical conductivity, calculated by the well-known equation: σ=l/(R∗S), where l is the electrode separation (i.e., the sample thickness), S is the electrode area, and R is the measured resistance (in Ω), showed very different behaviours for the h-BN-only and hybrid systems. As shown in Figure 10a, the electrical conductivity remained nearly constant across the h-BN series, with values on the order of a few pS/m. In contrast, graphite addition at fixed 20 wt% h-BN led to a progressive increase in conductivity, from approximately 4 pS/m up to approximately 650 pS/m (Figure 10b). Each electrical conductivity value was obtained as the average of three repeated measurements. The standard deviation was typically about 10% of the measured value. Because of the extremely low currents involved, these measurements are inherently sensitive to instrumental resolution, leakage currents, and environmental electromagnetic noise. To reduce external interference, the measurements were carried out inside a metallic enclosure providing partial shielding from the surrounding environment.

**Figure 11:**
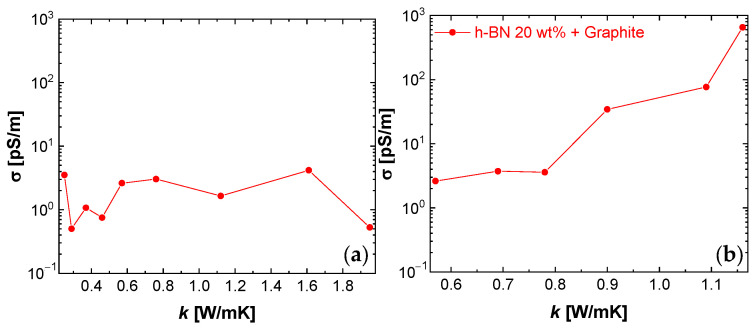
Electrical conductivity versus thermal conductivity for the h-BN series (**a**) and for the hybrid series at fixed 20 wt% h-BN with increasing graphite content (**b**).

A comparison between electrical and thermal conductivity is shown in Figure 11. No clear correlation was observed in the h-BN-only system, where substantial increases in thermal conductivity occurred without significant changes in electrical conductivity (see Figure 11a). By contrast, in the h-BN/graphite hybrids, thermal and electrical conductivity increased concurrently, indicating a coupled effect of graphite addition on charge and heat transport (see Figure 11b).

### 3.6. Mechanical Properties

**Figure 12:**
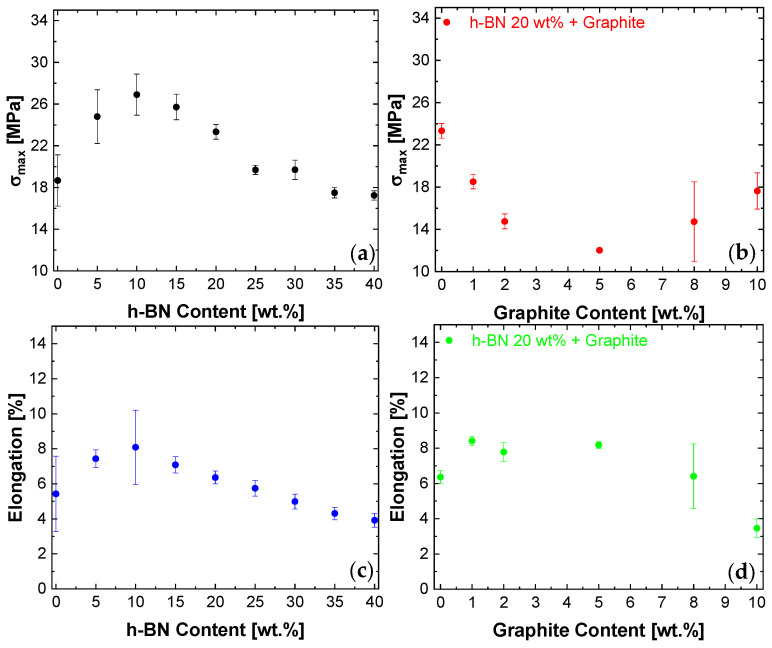
Maximum tensile stress as a function of h-BN content (**a**) and graphite content at fixed h-BN loading (20 wt%) (**b**); elongation at break as a function of h-BN content (**c**) and graphite content at fixed h-BN loading (20 wt%) (**d**).

The tensile results are summarized in Figure 12. For the h-BN-only series, the maximum tensile stress exhibited a non-monotonic trend: a slight increase was observed at low filler contents, followed by a gradual decrease at higher h-BN loading (Figure 12a). The elongation at break also decreased progressively with increasing h-BN content (Figure 12c), indicating a transition toward more brittle behavior.

In the hybrid series, graphite addition at a fixed 20 wt% h-BN led to a further decrease in maximum tensile stress (Figure 12b). The elongation at break remained nearly unchanged at low graphite loading and then decreased more markedly at higher graphite fractions (Figure 12d). Overall, the tensile results indicate that increasing filler content, especially in the presence of graphite, progressively reduces ductility and mechanical tolerance to deformation.

## 4. Discussion

The results show that the behaviour of the printed composites is governed by a coupled interplay among filler loading, formulation rheology, DLP processability, and microstructural organization. In this respect, the main contribution of the present work is not the achievement of record thermal conductivity, but the definition of a practical solvent-free DLP-compatible formulation window in which processability, structure, and functional properties can be systematically correlated.

The viscosity data highlight a first important trade-off. Increasing h-BN loading caused a strong monotonic rise in viscosity, while graphite addition at fixed h-BN content further shifted the formulations toward higher-viscosity regimes. This explains why compositions up to about 20 wt% h-BN remained relatively straightforward to print, whereas higher filler loadings and graphite-rich hybrids required tighter control of exposure and settling conditions. Therefore, viscosity should be considered a direct indicator of the practical processability window for DLP printing.

The TG/DTA results and microscopy observations indicate that the adopted processing route was sufficiently robust to avoid major filler sedimentation under the investigated conditions. At the same time, it should be noted that, for substantially longer print cycles, sedimentation of high-density fillers may become more relevant. In this respect, more industrial resin-printing systems may incorporate resin-mixing or recirculation strategies to preserve formulation homogeneity during printing. The OM and FE-SEM images revealed a generally uniform dispersion of the fillers and the expected 50 μm layered architecture produced by DLP printing, although occasional agglomerates and local pull-out features were also observed. These imperfections are consistent with a conductive network that is effective but still not fully optimized. From a mechanical standpoint, the observed deterioration in tensile strength and elongation at break with increasing filler loading can be interpreted as the result of multiple concurrent mechanisms. The incorporation of fillers into the resin alters the physical and chemical balance of the system. Although this enables a substantial increase in thermal conductivity relative to the neat matrix, a progressive deterioration of mechanical properties is observed with increasing filler concentration. In this context, h-BN agglomerates may act as local stress concentration sites, while two additional mechanisms may also contribute: filler–matrix debonding and possible interlayer weakening during DLP printing. The presence of fillers can locally affect both chemical adhesion and physical interactions with the photopolymer matrix, promoting micro-void formation and thereby reducing tensile properties. In addition, the fillers may limit curing degree and homogeneity because they can scatter and/or absorb UV light, an effect that is expected to be more pronounced for carbon-based fillers. Development of high-thermal-conductivity UV-photopolymerizable systems with mechanical properties closer to those of the neat matrix would likely require improved resin wetting and impregnation through filler surface modification and, in the case of 2D materials, exfoliation to obtain a more homogeneous dispersion in the matrix. From a thermal standpoint, h-BN-rich formulations provided the most effective route to increase through-thickness thermal conductivity while maintaining electrical insulation. The increase from approximately 0.25 W/mK for the neat resin to approximately 1.95 W/mK at 40 wt% h-BN demonstrates that substantial thermal enhancement can be achieved even without solvents or engineered filler architectures. The attained values are consistent with the largely random filler distribution observed in the present composites and reported for similar systems in the literature [1,5,10]. In particular, Li et al. [18] reported 1.51 W/mK at 40 wt% h-BN in a bismaleimide-based composite. In addition, Bagatella et al. [19] reported a through-thickness thermal conductivity of 0.69 W/mK for a 3D-printed BN-filled polymer composite containing 32 wt% BN, while Han et al. [20] reported a through-thickness value of 0.60 W/mK for an epoxy/BNNS composite containing 8 wt% BNNS. Therefore, the value of 1.95 W/mK achieved here at 40 wt% h-BN compares favorably with selected h-BN-filled polymer systems reported in the literature. A summary of representative literature values is provided in Table 2.

The hybrid h-BN/graphite systems provided additional but more moderate gains in thermal conductivity. The largest increases were observed at low graphite additions, whereas further additions produced diminishing returns. This suggests that graphite contributes to the development of supplementary heat-transfer pathways within the h-BN-containing matrix, but does not produce a radically more efficient conductive architecture. This behavior can also be interpreted in light of interfacial heat-transfer mechanisms commonly discussed in the literature for thermally conductive polymer composites [21,22]. In particular, interfacial thermal resistance and phonon scattering at filler–matrix and filler–filler junctions are recognized as important factors limiting heat transport, especially in systems containing fillers with different chemistry and morphology [21,22]. Within this framework, graphite may contribute to supplementary conductive pathways in the h-BN-containing matrix, but the coexistence of h-BN platelets and graphite flakes may also introduce additional interfacial complexity. Therefore, the moderate increase in thermal conductivity observed in the present hybrid systems suggests that, although hybrid network formation is promoted, the overall heat-transfer efficiency remains limited by imperfect interfacial contacts and scattering effects [21,22]. A more quantitative analysis of these mechanisms would require dedicated interfacial characterization and/or a specific theoretical model, which lies beyond the scope of the present work.

This interpretation is supported by the electrical-conductivity results. In the h-BN-only series, thermal conductivity increased substantially while electrical conductivity remained nearly unchanged, confirming that h-BN improves heat transport without creating electronic conduction paths. In contrast, graphite addition increased the electrical conductivity by orders of magnitude and generated a clear thermal/electrical correlation in the hybrid systems. It should also be noted that, in the pS/m range, the absolute conductivity values must be interpreted with caution because of the very high resistances involved and the greater sensitivity of the measurements to instrumental limitations, leakage currents, and environmental noise. In this sense, the measured values are mainly indicative of the persistence of insulating behaviour and of the relative trends across the formulation series, rather than highly precise absolute conductivity values. From an application standpoint, this means that h-BN-rich formulations are preferable when electrical insulation is required, whereas h-BN/graphite hybrids may be useful when coupled thermal/electrical functionality is desirable.

The phenomenological percolation-type model reproduced the thermal-conductivity trend of the h-BN series reasonably well. In particular, the fitted transition parameter should be regarded as a descriptor of the observed sigmoidal trend rather than as a strict geometrical percolation threshold. Its relatively high value is consistent with the fact that effective through-thickness heat-transfer pathways require not only the presence of h-BN platelets, but also sufficiently efficient platelet–platelet contacts across the sample thickness. This condition is hindered by incomplete inter-particle contact, limited effective contact area, interfacial thermal resistance, local agglomeration, random platelet distribution, and possible microstructural discontinuities associated with the layer-by-layer DLP process. Similarly, the Hashin–Shtrikman-based interconnectivity index is useful for comparing relative pathway development across compositions and for supporting the interpretation of progressively more effective heat-transfer pathways at increasing filler content. Consistently, the interconnectivity index reaches ~0.7 at 40 wt% h-BN, indicating that the conductive network becomes significantly developed at the highest filler loading, although it does not yet reach the ideal upper-bound condition. The model is therefore intended to describe and predict the evolution of thermal conductivity as a function of h-BN concentration under the present printing conditions, whereas its extension to different printing parameters or layer thicknesses would require dedicated validation. This interpretation is also qualitatively consistent with literature reports on comparable h-BN/polymer composite systems, in which efficient thermal transport is generally achieved only at relatively high filler loading when heat conduction remains limited by incomplete filler contact, interfacial thermal resistance, and random platelet organization [1,3,18,23,24].

In the present study, we focused on through-thickness thermal conductivity because this direction is especially relevant for the intended thermal-management function of the printed parts and because the experimental setup and specimen geometry were specifically designed for through-thickness TPS measurements. At the same time, it should be acknowledged that platelet-like fillers such as h-BN and graphite may induce anisotropic thermal transport behaviour in polymer composites, since their processing-induced orientation can significantly affect the balance between in-plane and through-plane heat conduction [3,25]. In the present work, however, our objective was not to control or optimize filler orientation, but rather to investigate the thermal-conductivity enhancement achievable through a simple and practical DLP-printing approach using unmodified fillers and commercially accessible processing conditions. In this sense, the aim was to evaluate the potential and the limitations of this straightforward formulation strategy before introducing additional variables related to filler alignment or orientation-assisted processing.

Finally, the tensile results confirm that thermal and electrical gains are accompanied by reduced ductility and, at high filler loading, by lower tensile strength. These changes are consistent with the increasing presence of rigid platelet fillers, local agglomerates, and interfacial or structural discontinuities. In addition, because increasingly high filler loading required composition-dependent adjustment of the printing parameters, including longer exposure times, a higher number of bottom layers, and reduced build-plate lift speed, in order to maintain printability and achieve fully cured specimens, a contribution from reduced interlayer integrity cannot be excluded. In the present work, what could be clearly discriminated was whether a given formulation and parameter set led to successful curing and fabrication of acceptable specimens or, conversely, to printing failure. By contrast, intermediate levels of curing quality or interlayer consolidation that may contribute to progressive mechanical degradation without causing evident print failure could not be resolved within the scope of the present study. Although interlayer adhesion energy was not measured directly in the present work, the observed decrease in elongation at break and, at high loading, in tensile strength is consistent with the combined effects of filler-induced embrittlement, local agglomeration, possible filler–matrix debonding, and possible weakening of layer-to-layer cohesion under increasingly demanding curing conditions. This interpretation is also consistent with previous studies on additively manufactured layered composites, where interlayer adhesion was shown to depend significantly on process parameters such as layer height [26]. No chemical surface modification was applied to either h-BN or graphite in the present work. Therefore, the observed agglomeration behaviour must be interpreted in the context of unmodified fillers dispersed only by the adopted mixing route. Previous literature shows that surface modification of boron nitride can reduce agglomeration and improve filler–matrix compatibility, with possible benefits for both thermal and mechanical performance [27,28]. For example, Liu et al. [27] reported that silane-modified BN nanosheets exhibited improved dispersion and reduced agglomeration compared with unmodified h-BN in UV-cured polyacrylate systems. However, the effect of surface modification is not purely beneficial and depends strongly on the chemistry and structure of the grafted interfacial layer, which can also influence heat transport [28]. Therefore, although surface modification is a promising strategy to improve dispersion, its systematic evaluation lies beyond the scope of the present work and may be addressed in future studies. Although the formulation containing 40 wt% h-BN exhibited a through-thickness thermal conductivity nearly one order of magnitude higher than that of the neat resin, further improvement is expected to depend strongly on achieving better boron nitride dispersion, particularly in the form of exfoliated sheets, in order to maximize the benefits of the intrinsic thermal properties of h-BN. Overall, the present results show that the design of thermally enhanced DLP-printable composites is governed by trade-offs among printability, thermal transport, electrical behavior, and mechanical integrity.

From a practical standpoint, the marked reduction in elongation at break and the progressive decrease in tensile strength at high filler loading substantially narrow the realistic application window of the present materials. In particular, these composites are not suitable for applications requiring high conformability, high deformability, or significant strain tolerance, such as soft thermal interface pads or gap fillers. Rather, they are more appropriately positioned as self-supporting, rigid or semi-rigid, electrically insulating components for thermal management operating under low mechanical loads. This interpretation is supported by both literature and commercial benchmarks. For example, Bagatella et al. [19] demonstrated the use of BN-filled 3D-printed composites for lightweight heat sinks in electronic thermal management; in that study, the composite containing 32 wt% BN exhibited a strain at break of 3.4–4.5%, depending on print orientation, indicating that limited ductility can still be compatible with rigid thermally functional printed parts. Their reported through-thickness thermal conductivity was 0.69 W/mK, which is lower than the 1.95 W/mK achieved in the present work at 40 wt% h-BN. In addition, representative commercial rigid thermally conductive and electrically insulating plastics show similarly low or even lower ductility: CoolPoly^®^ D3612 exhibits a strain at break of 0.68% [29], while LNP KONDUIT OX11314 shows a strain at break of 0.60% [30]. Commercial application notes for this class of materials identify use in electronics, power electronics, motors, connectors, lighting, inverter housings, and motor insulators. Therefore, the present DLP-printed composites are better positioned as materials for customized rigid thermal-management components, such as housings, covers, insulating inserts, or low-stress heat-spreading parts, rather than as compliant thermal interface materials.

More broadly, the present results highlight the intrinsic trade-off of this approach: the addition of thermally conductive fillers is necessary to enhance heat transport in DLP-printable UV-curable resins, but it also affects other key properties of the formulations and printed parts, including curability, rheology, and mechanical performance. Future work will therefore focus on mitigating the deterioration of the mechanical properties while preserving the thermal enhancement.

## 5. Conclusions

Solvent-free UV-curable composites filled with h-BN and h-BN/graphite hybrids were successfully formulated and processed by DLP 3D printing using commercially available equipment. The results show that filler loading strongly affects resin viscosity and therefore the practical printing window, but reliable fabrication remained achievable up to 40 wt% h-BN through composition-dependent adjustment of the build parameters. Thermal analysis and microscopy confirmed that the processed parts exhibited filler contents consistent with the nominal formulation and showed generally uniform filler dispersion together with the expected layered DLP architecture.

The through-thickness thermal conductivity increased from approximately 0.25 W/mK for the neat resin to approximately 1.95 W/mK at 40 wt% h-BN, demonstrating that substantial thermal enhancement can be achieved without solvents or deliberately engineered filler architectures. At a fixed 20 wt% h-BN, graphite addition produced a further but more moderate increase in thermal conductivity, up to approximately 1.16 W/mK, while simultaneously increasing electrical conductivity. These results indicate that h-BN-rich formulations are the most effective option for improving thermal transport while preserving electrical insulation, whereas h-BN/graphite hybrids are better understood as systems that enable tunable coupled thermal/electrical behaviour.

The phenomenological percolation-type model captured the composition-dependent thermal-conductivity trend of the h-BN series and, together with the Hashin–Shtrikman-based interconnectivity analysis, supported the interpretation of progressively more effective heat-transfer pathways at increasing filler content.

Mechanical testing showed that the thermal and electrical gains are accompanied by reduced ductility and, at high filler loading, by lower tensile strength. Accordingly, the design of these DLP-printable composites is governed by a balance among processability, thermal performance, electrical response, and mechanical integrity. In this framework, the present study provides a practical process–structure–property basis for the development of thermally enhanced UV-curable composites for DLP-based additive manufacturing applications.

## Figures and Tables

**Table 1:** Nominal filler content and TGA residual mass measured at 900 °C for the neat resin, h-BN-filled composites, and hybrid h-BN/graphite formulations.

Sample	Filler Content [wt%]	TGA Residual Mass @900 °C [wt%]
Resin	0	0.15
Resin + 5 wt% h-BN	5	4.75
Resin + 25 wt% h-BN	25	24.69
Resin + 20 wt% h-BN + 1 wt% Graphite	21	20.74
Resin + 20 wt% h-BN + 2 wt% Graphite	22	21.34
Resin + 20 wt% h-BN + 5 wt% Graphite	25	24.63
Resin + 20 wt% h-BN + 8 wt% Graphite	28	27.15

**Table 2:** Comparison of the through-thickness thermal conductivity achieved in the present work with selected h-BN-filled polymer systems reported in the literature.

Matrix/System	Processing Method	BN Loading	Thermal Conductivity (W/mK)	Ref.
UV-curable acrylate resin	DLP printing	40 wt% h-BN	1.95	This work
Bismaleimide composite	Casting + thermal curing	40 wt% h-BN	1.51	[18]
BN-filled polymer composite	FDM/3D printing	32 wt% BN	0.69	[19]
Epoxy/BNNS composite	Vacuum-assisted resin infusion + thermal curing	8 wt% BNNS	0.60	[20]

## Data Availability

The original contributions presented in this study are included in the article. Further inquiries can be directed to the corresponding authors.
